# An Investigation of the Fracture Loads Involved in the Framework of Removable Partial Dentures Using Two Types of All-Ceramic Restorations

**DOI:** 10.3390/biomimetics8010113

**Published:** 2023-03-09

**Authors:** Fahad Hussain Alhamoudi, Lujain Ibrahim N. Aldosari, Abdulkhaliq Ali F. Alshadidi, Saeed Awod Bin Hassan, Maram Ali M. Alwadi, Sunil Kumar Vaddamanu, Marco Cicciù, Giuseppe Minervini

**Affiliations:** 1 Dental Technology Department, College of Applied Medical Science, King Khalid University, Abha 61421, Saudi Arabia; 2 Department of Prosthetic Dentistry, College of Dentistry, King Khalid University, Abha 61421, Saudi Arabia; 3 Department of Restorative Dental Sciences, College of Dentistry, King Khalid University, Abha 61421, Saudi Arabia; 4 Dental Health Department, College of Applied Medical Science, King Saud University, Riyadh 11451, Saudi Arabia; 5 Department of General Surgery and Medical-Surgical Specialties, School of Dentistry, University of Catania, 95124 Catania, Italy; 6 Multidisciplinary Department of Medical-Surgical and Odontostomatological Specialties, University of Campania “Luigi Vanvitelli”, 80121 Naples, Italy

**Keywords:** removable partial denture, ceramic crowns, fracture strength

## Abstract

Retention and support are needed for removable partial denture stability. The stability can be achieved by clasps, occlusal and cingulum rests on healthy abutment teeth. However, implants or crowns can be used to support the removable partial denture instated on unhealthy abutment teeth. This study was conducted to investigate the fracture strength of two types of all-ceramic restorations used as abutments for the removable partial denture framework. The crowns were manufactured with two types of ceramic materials: zirconia and IPS e.max Press ceramics. The metal alloy (cobalt-chrome) was cast to form the removable partial denture framework. A universal testing machine was used to evaluate the fracture strength of both ceramic crown materials. The results presented no fractures in all-ceramic crowns, but deformation of the partial denture frameworks occurred. With the limitation of this study, it can be concluded that zirconia and IPS e.max Press ceramic can be used as abutments to provide adequate support to the removable partial denture.

## 1. Introduction

Partially dentate problems are still present in dental clinical practices. An increase in these problems means that, over time, patients may lose more than one tooth and become partially dentate. These scenarios will generally increase the demand for dental prosthodontics [1]. This includes the most popular prostheses for elderly patients—removable partial dentures (RPDs). Implants, bridges, and RPDs are potential options to restore missing teeth for the partially dentate patient [2,3,4,5]. However, due to the age and periodontal condition of the patient such as gingivitis and periodontitis, RPDs are the most suitable choice for treatment [6].

The patient’s age can affect the success of the dental implant treatment. The elderly have less healing time due to the decreased formation of new bone around an implant [7]. Therefore, dental implants have yet to be considered a suitable treatment for everyone [8]. The human jaw could be affected by osteoporosis [9]. Due to the composition of the maxilla, which contains more trabecular bone than the mandible, the maxilla jaw is more likely to be affected by osteoporosis than the mandible jaw [10]. Considering all these factors, RPD is the most common treatment option [6].

Despite the effect of the RPD on the periodontal condition and other problems, for instance, pain in the soft tissues, especially with free-end saddles [11], RPD is still one of the treatment options in clinical practice to resolve missing teeth problems [12]. Some principles must be applied when constructing RPDs, such as retention and support to resist oral forces and enhance denture stability. These can be achieved by incorporating occlusal, cingulum rest seats and clasps. This process requires a tooth in good condition to apply these rest seats. However, due to the condition of the abutment teeth, which may prevent these seats from being incorporated, implants or crowns may be used to achieve the benefits of the rest seats and support the RPD framework [13]. Different studies have discussed using implants and crowns as an abutment for the RPD framework [11,14,15]. Crowns could be fitted as an abutment by using cast metal or porcelain-fused-to-metal (PFM) crowns, which have been considered acceptable results due to strength and longevity factors [16]. Alternatively, and for aesthetic reasons, crowns could be fabricated using all-ceramic materials and act as an abutment to support the RPD framework [17].

Due to the disadvantages such as color and biocompatibility, the demand for aesthetic materials such as all-ceramics is increasing. Despite dental ceramic materials’ high performance and strength, fractures are commonly reported with this type of restoration [18]. Clinical complaints are still presented regarding all-ceramic restoration fracturing [19], while most complaints in clinical practice are about fracturing of all-ceramic restorations in the posterior region [20]. Even with a comparison between PFM and all-ceramic restoration, a systematic review by Sorrentino et al. [21] concluded that all-ceramic restorations showed lower survival rates than PFM restorations, which indicates how the type of restoration can affect the success of supporting the RPD framework. Fracturing of all-ceramic restorations could be caused by occlusal and mastication forces created by patients [22,23]; for this reason, all-ceramic restorations are more suitable for restoring the anterior teeth and are still debatable to be used in the posterior region [24]. Further, Rekow et al. [25] suggested that, due to the complaints about all-ceramic restoration fracturing, the strength of these types of restoration is insufficient for long-term service in posterior teeth.

In addition, fracturing of all-ceramic restorations can be caused by a load of biting forces exerted, particularly in the posterior areas [26]. Numerous studies have investigated load forces in the patient’s mouth, and greater load forces were observed in the posterior areas. Other studies investigated the biting forces’ mean value and standard deviation [27,28,29]. On the other hand, the load forces in the patient’s mouth are different when dental prostheses are involved [30]. For instance, Miyaura et al. [31] reported that biting forces for people with full metal crowns are 333.2 ± 234.1 N, for people with bridges are 323.8 ± 236.3 N and for people with RPDs are 140.5 ± 126.4 N. From previous studies, the mean value and standard deviation of the mastication and biting forces exerted in the patient’s mouth are theoretically lower than the mean value and standard deviation of all-ceramic restoration materials. In addition, the results are significant in comparison between the mean value and standard deviation. In comparing fracture strength between PFM and all-ceramic restoration, Rao and Chowdhary [32] observed 4736.5 ± 2267.6 N for zirconia, 1566 ± 505.7 N for IPS Empress and 3275.7 ± 468 N for PFM restorations. Most of these results have been achieved by applying the load forces directly to the tooth during the test of the samples.

Limited previous studies were found to investigate the fracture strength of all-ceramic restorations for removable partial dentures. For this reason, this study aimed to investigate and compare the fracture strength of two selected types of all-ceramic restorations involved in the removable partial denture framework.

## 2. Materials and Methods

Computer-Aided Design/Computer-Aided Manufacturing (CAD/CAM) was used to mill the final restoration, such as zirconia samples or wax with IPS e.max Press and RPD samples, to standardize the sample fabrication and dimension [26,27,28,29,31,33].

### 2.1. Model Fabrication

**Figure 1:**
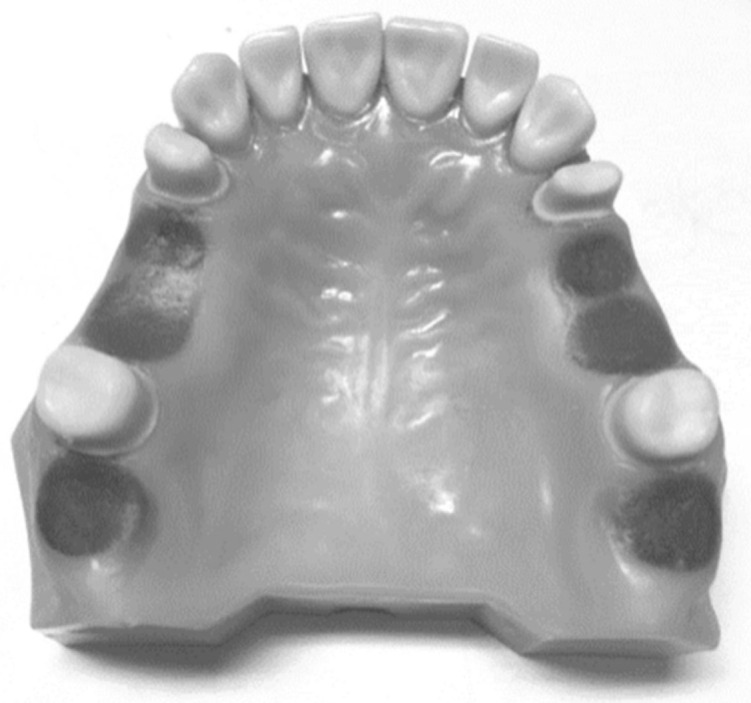
The selected model case for the study.

A unilateral edentulous space with natural teeth remaining in both anterior and posterior models, known as class III modification 1, was selected for this study (Figure 1). The selected model was duplicated as a dental stone model using a mold made of silicon material.

**Figure 2:**
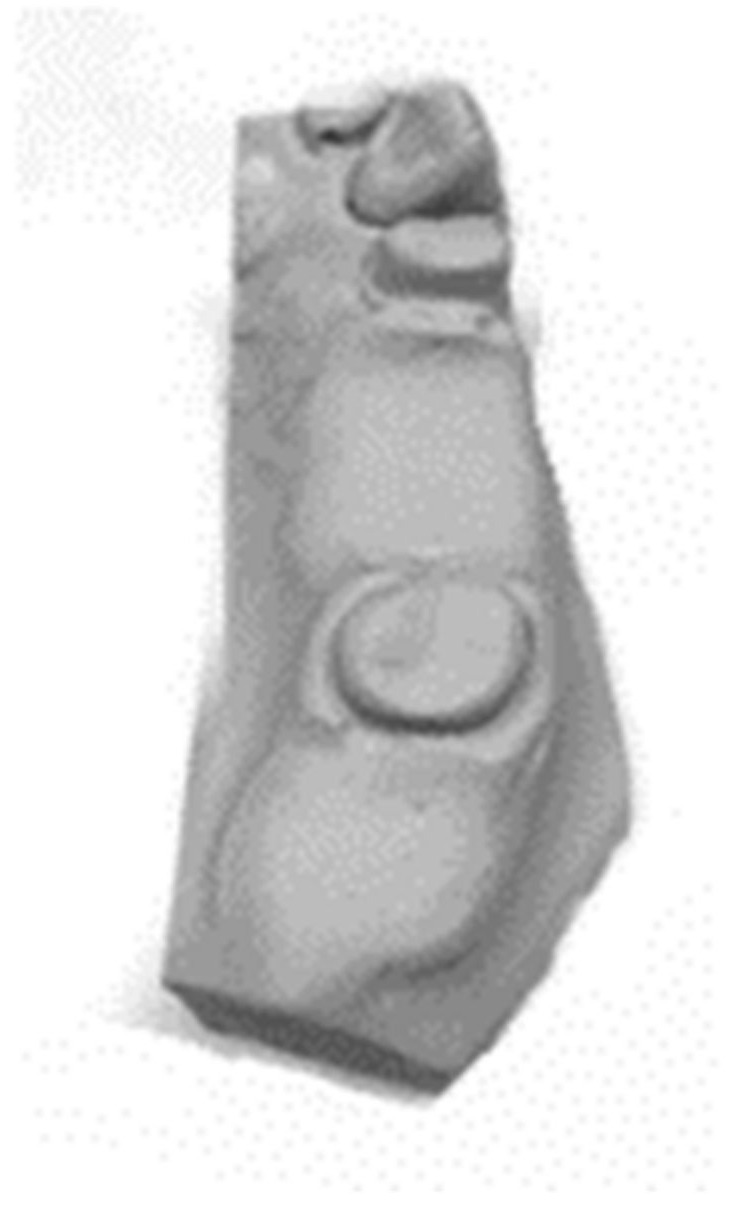
The model targeted area for the study.

The duplicated model was trimmed until it reached the target area, the edentulous area between two prepared teeth (distally for 1st premolar and mesially for the 2nd molar). (Figure 2). The selection of these specific teeth was according to the previous research [26,33]. After the investigation of the occlusal and biting forces, it was found that the most forces were recorded in the posterior region and particularly in the first premolars and molars, which have been recorded at the range between 481.6 ± 1000 N for both males and females without any dental prosthodontics involved [27,28,29]. However, involving different dental prosthesis types, the range was between 140.5 ± 333.2 [31]. This targeted area was then duplicated as a master resin model using the same technique mentioned above. The resin material was selected to mimic the modulus elasticity of human dentition. The master model was scanned using 3D scanner to create a digital form which was used to design and produce the RPD and crowns.

### 2.2. Crown Fabrication

The crowns were designed on the digitally scanned model with these measurements (thicknesses 1.2 mm, chamfer 1 mm deep, convergence 6 degrees, occlusal rests preparation 1 mm (distally for the first premolar and mesially for the second molar)) using Amman Girrback Ceramill 600 software. Two ceramic materials were used to fabricate all-ceramic crowns (IPS e.max Press lithium disilicate glass ceramics and zirconia).

For the IPS e.max empress, the designed crowns were milled into the wax using the CAM process (milling manufacturing). The wax crowns were then sprued, invested, cast, divested, finished and glazed according to the manufacturer’s instructions for the IPS e.max Press ceramic materials (IvoclarVivadent, Koblach, Austria).

For zirconia materials, the designed crowns were milled into the zirconia using the CAM process (milling manufacturing), and the zirconia crowns were sintered according to the manufacturer’s instructions.

All IPS e.max Press and zirconia crowns were cemented into the resin models using 3M ESPE Relyx luting Cement and scanned for the RPD framework (saddle and occlusal rest area) design.

### 2.3. RPD Framework Fabrication

**Figure 3:**
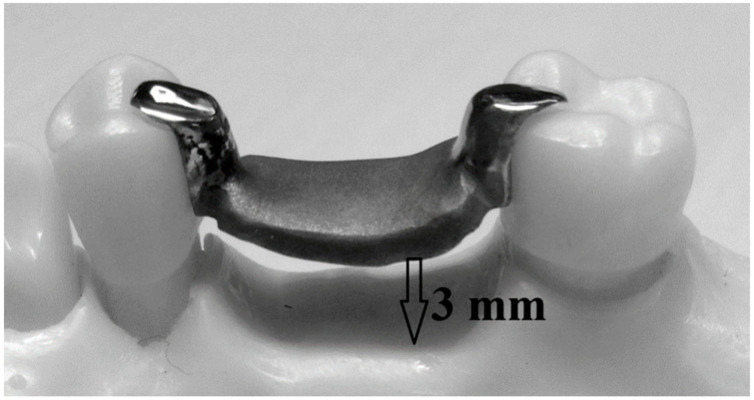
The created space between the saddle and alveolar ridge is 3 mm.

The RPD framework was designed using the digitally scanned model with the default thickness of the saddle and occlusal rests area (thicknesses 1.2 mm and occlusal rest thinness preparation 1.2 mm (distally for the first premolar and mesially for the second molar)) using Amman Girrback Ceramill 600 software. Moreover, a distance of approximately 3 mm was created between the saddle and the alveolar ridge to allow the universal testing machine crosshead to be generated when the load is applied (Figure 3). The designed RPD was milled into the wax using the CAM process (milling manufacturing). The wax crowns were then sprued, invested, cast, divested, finished and polished according to the manufactures instruction for BEGO (Wironit, Bremen, Germany) cobalt-chrome alloy materials. The RPDs were ready to be fitted into the crowns.

All the samples were subjected to the test by a universal testing machine (Admet, Lloyd LRX, Largo, FL, USA). A single fracture load was applied at a rate of 1 mm/min to investigate the fracture strength of both ceramic crown types (IPS e.max Press ceramics and zirconia) for RPDs.

### 2.4. Samples Summery

**Figure 4:**
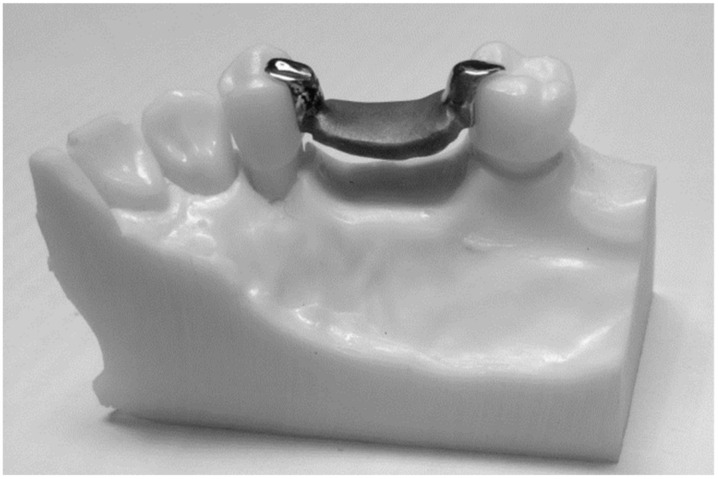
The final design of the sample.

Twenty samples were fabricated; each had two all-ceramic crowns connected by the removable partial denture saddle framework, with two occlusal rest seats on the abutment crowns merged distally in the first premolar and mesially in the second molar (Figure 4 and Table 1).

## 3. Results

Ten samples from both groups (IPS e.max Press and zirconia) were subjected to the test by a universal testing machine to compare each group’s mean fracture strength. The results showed no fracturing of ceramic crowns for both groups after applying a single cycle of fracture strength test at a 1 mm/min rate, which prevented the results from being recorded, and these result were excluded.

**Figure 5:**
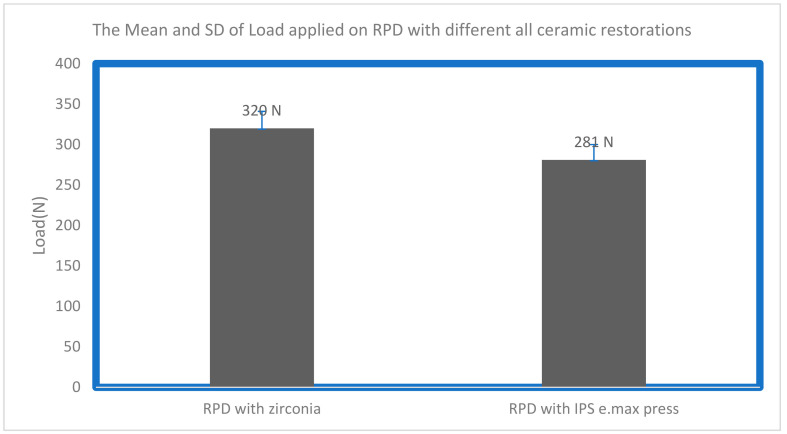
The mean and standard deviation of fracture strength of RPD (Co-Cr alloy) used with all-ceramic crowns (IPS e.max Press and zirconia materials).

For the RPD saddles with both ceramic crowns, a deformation was observed. This could be related to the differences in design and weak mechanical properties (modulus of elasticity and tensile strength) of Co-Cr compared to all-ceramic restorations (Table 2 and Figure 5).

**Figure 6:**
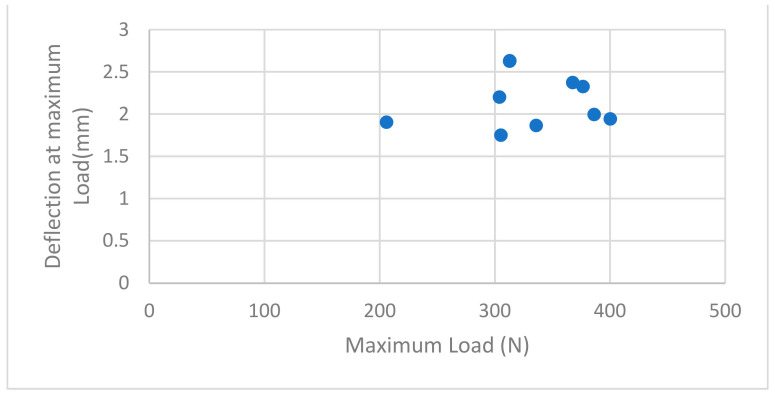
Maximum deflection with maximum load applied to zirconia crowns with RPD framework.

The maximum load of the RPD framework with the zirconia group was at 400 N with a maximum deflection of 1.9409 mm. However, the minimum load was at 206 N with a maximum deflection of 1.9014 mm (Figure 6).

**Figure 7:**
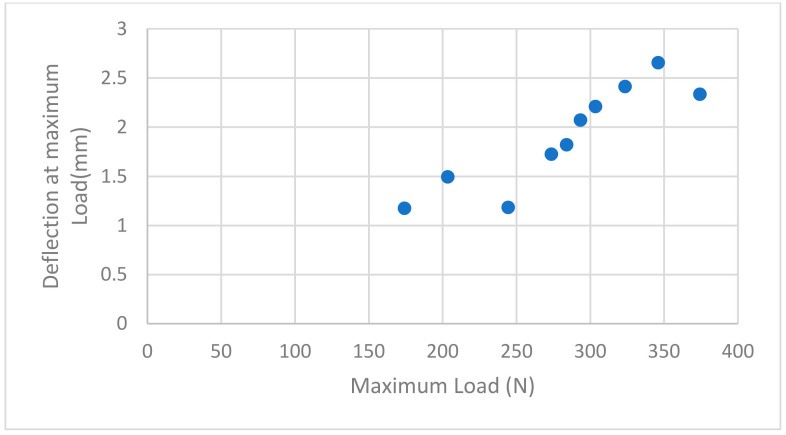
Maximum deflection with maximum load applied to IPS e-max Press crowns with RPD framework.

For the RPD framework with the IPS e.max Press group, the maximum load was at 374 N with a maximum deflection of 2.3335 mm, while the minimum load was at 174 N with a minimum deflection of 1.1745 mm (Figure 7).

**Figure 8:**
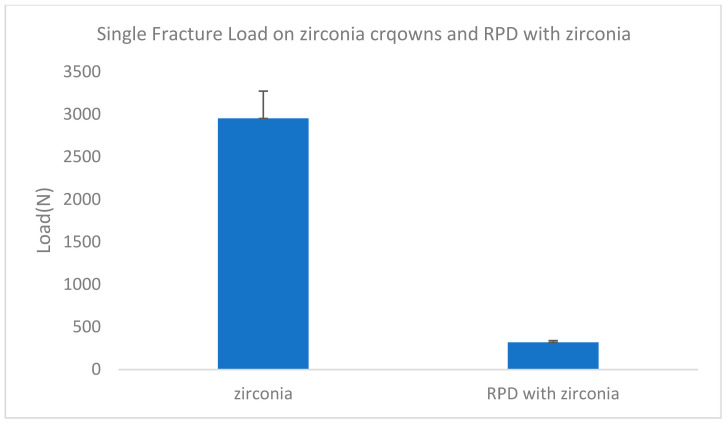
The mean fracture strength of zirconia crowns from the previous research [34] was significantly higher than that of RPD saddles with zirconia crowns in this study (*p* ≤ 0.05).

The mean fracture strength of zirconia crowns from the previous research [34] was significantly higher than that of RPD saddles with zirconia crowns in this study (*p* ≤ 0.05) (Table 3 and Figure 8)

The mean fracture strength of zirconia crowns from the previous research [31] was significantly higher than that of RPD saddles with zirconia crowns in this study (*p* ≤ 0.05) (Table 3). This indicated the possibility of using zirconia crowns to support RPD and as restoration for the posterior teeth (Figure 7).

**Figure 9:**
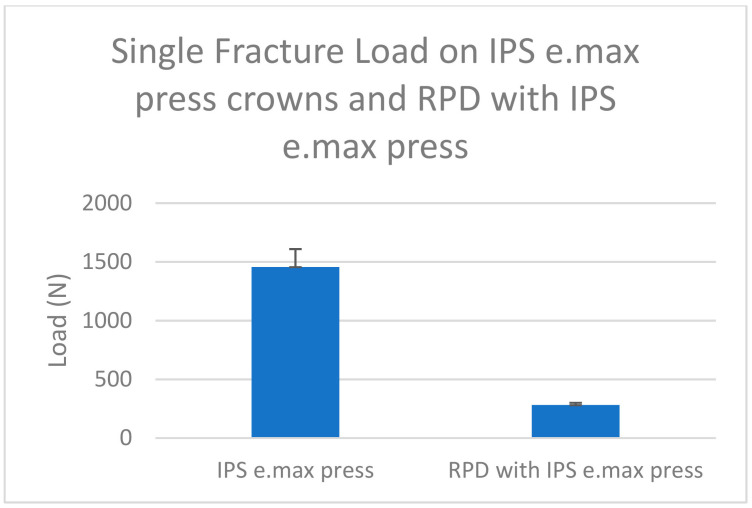
The mean fracture strength of IPS e.max Press crowns from the previous research [32] was significantly higher than that of RPD saddles with IPS e.max Press crowns in this study (*p* ≤ 0.05).

The mean fracture strength of IPS e.max Press crowns from the previous research [32] was significantly higher than that of RPD saddles with IPS e.max Press crowns in this study (*p* ≤ 0.05) (Table 4). This indicated the possibility of using IPS e.max Press crowns to support RPD and as restorations for the posterior teeth (Figure 9).

## 4. Discussion

This study was carried out to investigate and compare the fracture strength of two types of ceramic restorations involved in the RPD framework and determine which type of ceramic restoration can be used to support the RPD framework.

**Figure 10:**
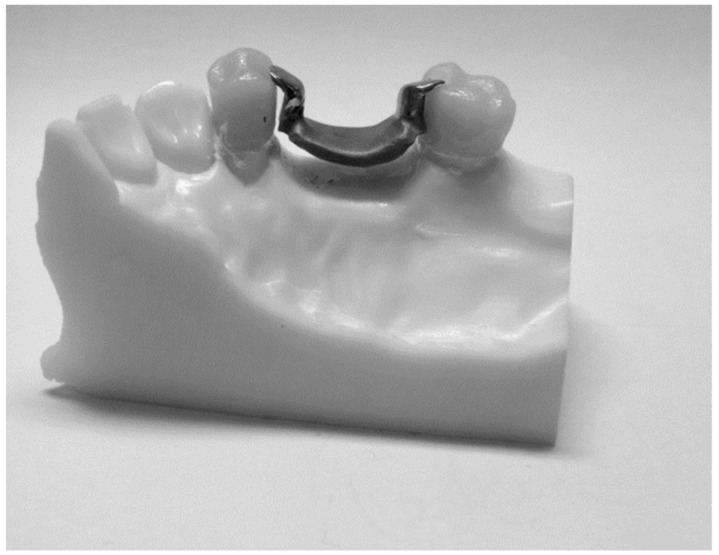
Sample after loading was applied.

Twenty samples were fabricated and divided into two groups: group A zirconia crowns and group B IPS e.max Press crowns. The CAD/CAM technology was used to fabricate all crowns with similar dimensions according to the previous study by Jang et al. [34,35] with a thicknesses of 1.2 mm, chamfer of 1 mm deep, convergence of 6 degrees, occlusal rests preparation of 1 mm. Due to the adequate strength of both ceramic materials, the Co-Cr saddle was deformed while applying the fracture load (Figure 10) [36]. Therefore, the fracturing of ceramic restorations was impossible, but this can be modified in future studies by modifying crown dimensions as the materials’ thickness impacts the fracture strength. Jang et al.’s [34] study recorded different fracture strengths using zirconia materials, which were classified into five groups: group 1 was 2359 N for crowns with 0.5 mm, group 2 was 3216 N for crowns with 1.0 mm, group 3 was 3898 N for crowns with 1.5 mm. However, for groups 4 and 5 (2 mm and 2.5 mm), the results could not be measured due to the strength of those materials leading to the resin model being broken.

A variety of studies have investigated all-ceramic restorations under the fracture fatigue resistance or until fractures occurred [32,37,38], yet none or limited studies investigated the connection between all-ceramic restorations and RPDs as reported in this study. The fracture test in this study was conducted by applying the fracture load to the saddle area which is connected to all-ceramic crowns by the occlusal rests. It was a single fracture load at a rate of 1 mm/min for each sample. However, this can be modified in future studies by performing a cycle test to simulate the survival rate and involving other RPD components such as clasps and cingulum rests. Those modifications may have a different outcome for all-ceramic restorations.

**Figure 11:**
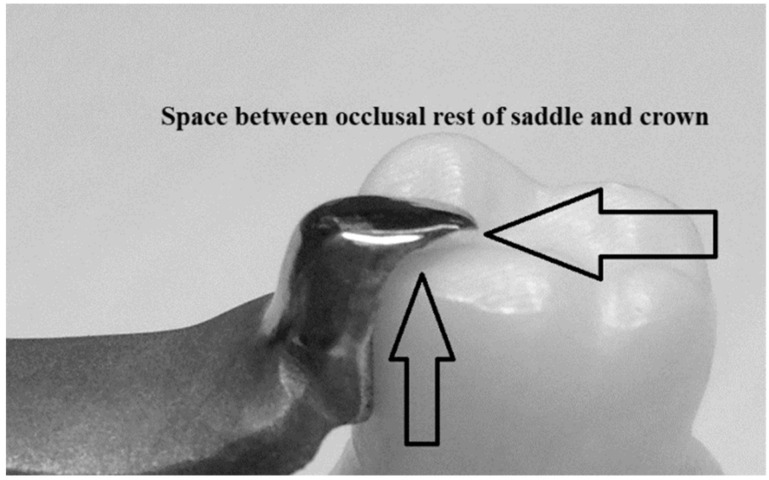
IPS e.max crowns with unfitted occlusal rest of RPD on the prepared area, space between occlusal rest and crown.

One factor that affects fracture test performance is the firmly located samples in the testing machine during the test. One of the IPS e.max Press crowns failed the test due to the improbably located occlusal rest, which led to the move and sliding of the RPD saddle during the test. That was not expected, as all samples were designed and fabricated using CAD/CAM. It was assumed that the RPD saddles would be accurate enough to obtain acceptable results. This assumption was related to the findings of a previous study, indicating that the adaptation of CAD/CAM fabricated dentures would be more accurate compared to the conventional methods [39]. It is worth mentioning that this was only noticed with one sample with IPS e.max crowns. This might be due to the fabrication process of the IPS e.max crowns via CAD/CAM producing wax crowns then pressed as the ceramic crown, or the excessive use of a glazing agent, which changed the dimension and geometrical shape of the fossa, occlusal rest or crown, causing improbable fitting of the RPD rest and saddle (Figure 11). Moreover, using a saddle with only an occlusal rest could be another drawback of the samples and study, as other components can improve denture retention and stability, such as the clasp or connectors.

All saddles in this study were designed by the Amman Girrback Ceramill software. The designed saddle and occlusal rest area dimensions were as follows: thicknesses of 1.2 mm and occlusal rest thinness preparation of 1.2 mm (distally for the first premolar and mesially for the second molar). The strength of any material may be affected by modified dimensions and design applied to the RPD and Co-Cr component, particularly for the cingulum and occlusal rest seats. This was confirmed in Sato et al.’s [40] report on thickness and shape impact on the designed occlusal rest’s fatigue strength. The study reported that the strength of the occlusal rest was increased with the increase in thickness. Furthermore, Sato et al. [40] provided some recommendations for the occlusal rest dimensions (such as a length of 3 mm, width of 3 mm and thickness of 1.0 mm) and designs (such as avoiding the sharp or over-rounded line angles).

Co-Cr alloy was used and selected for the RPD framework in this study because of its biocompatibility, mechanical and physical properties. Furthermore, Co-Cr alloy is the most used and affordable material for the RPD framework. The maximum fracture load was recorded on the RPD with the zirconia group at 400 N, while the minimum was with IPS e.max Press at 174 N. This can be related to the difference in mechanical strength between the ceramics used in this study (zirconia and IPS e.max Press).

Furthermore, the alloy material type and composition impact the outcome result according to Gapido et al.’s [41] study on the fatigue resistance between Ag-Pd-Cu-Au and Co-Cr as RPD materials. Their study indicated that Co-Cr alloys are more rigid and have greater ability to resist fatigue forces than Ag-Pd-Cu-Au alloys. Another study by Wu et al. [42] reported using Ticonium Premium alloy and Thermoflex acetyl resin as direct retainers for RPDs. Their study presented more significant deformation with acetyl resin than with Ticonium Premium alloy after 3 years of simulated use. A recent study investigated polyetheretherketone (PEEK) as a clasp material for RPD compared to Co-Cr. The results indicated that PEEK clasps had significantly less deformation in the fitting surface and approximately the same retentive force as Co-Cr clasps, suggesting the use of PEEK for aesthetic clasps for the RPD framework. Therefore, further studies can be applied to different RPD materials in future [41,42,43].

Lastly, the fracture test in this study was applied to the saddle area between the first premolar and second molar, which have been selected due to the maximum forces found in these regions according to the previous study. However, the outcomes might be changed using anterior teeth because of the different masticatory forces [44,45,46,47,48]. This led to the current and continued debate about whether to use all-ceramic restorations in the anterior or posterior teeth. A study indicated that all-ceramic restorations are more successful in anterior teeth than posterior [49] because the ceramic materials lack the necessary strength and the results of using all-ceramic restorations to restore posterior teeth are unpredictable [50]. However, the current dental ceramic materials, such as zirconia, have a significate mechanical strength for restoring posterior teeth. This debate might be reaching the end. Therefore, including both anterior and posterior teeth in the test may provide different outcomes for the benefit of the patient, dentistry and science.

## 5. Conclusions

With the limitation of this study, it can be concluded that all-ceramic restorations (zirconia and IPS e.max Press crowns) can support the framework of removable partial dentures, confirming this study’s hypothesis. Further studies of the all-ceramic restorations’ ability to support the removable partial denture framework of different materials and design is required.

## Figures and Tables

**Table 1:** The samples quantity, materials and types.

Item	Type of Material	Quantity
models	Model resin	20
crowns	30 zirconia	20
crowns	30 IPS e.max Press	20
RPD frameworks	Chrome-cobalt alloy	20

**Table 2:** The recorded values, mean and standard deviation of fracture strength of RPD (Co-Cr alloy) used with all-ceramic crowns (IPS e.max Press and zirconia materials).

Sample No	RPD with Zirconia	RPD with IPS e.max Press
1	335 N	284 N
2	305 N	273 N
3	386 N	244 N
4	313 N	346 N
5	400 N	374 N
6	367 N	323 N
7	376 N	174 N
8	206 N	203 N
9	312 N	293 N
10	304 N	303 N
mean	320 N	281 N
Standard deviation	±21 N	±19 N

**Table 3:** The mean fracture strength of zirconia crowns from the previous research [34] was significantly higher than that of RPD saddles with zirconia crowns in this study (*p* ≤ 0.05).

Sample No	Zirconia	RPD with Zirconia
1	3020 N	335 N
2	2949 N	305 N
3	3405 N	386 N
4	3353 N	313 N
5	3275 N	400 N
6	3277 N	367 N
7	2248 N	376 N
8	2402 N	206 N
9	2427 N	312 N
10	3184 N	304 N
mean	2954 N	320 N
Standard deviation	±320 N	±21 N

**Table 4:** The mean fracture strength of IPS e.max Press crowns from the previous research [32] was significantly higher than that of RPD saddles with IPS e.max Press crowns in this study (*p* ≤ 0.05).

Sample No	IPS e.max Press	RPD with IPS e.max Press
1	1593 N	284 N
2	2080 N	273 N
3	1378 N	244 N
4	1350 N	346 N
5	730 N	374 N
6	2265 N	323 N
7	955 N	174 N
8	1295 N	203 N
9	1123 N	293 N
10	1788 N	303 N
mean	1456 N	281 N
Standard deviation	±153 N	±19 N

## Data Availability

Data sharing is not applicable to this article.

## Abbreviations

RPD	Removable partial Denture
N	Newtons
Co-Cr	Cobalt-chromium
CAD/CAM	Computer-aided design/computer-aided manufacturing
(PEEK)	Polyetheretherketone
